# Using External Joint Stabilizer – Elbow (EJS-E) for treating elbow instability—biomechanical assessment and clinical outcomes

**DOI:** 10.1186/s12891-022-06103-0

**Published:** 2022-12-29

**Authors:** Ching-Hou Ma, Chin-Hsien Wu, Yen-Chun Chiu, Kun-Ling Tsai, I-Ming Jou, Yuan-Kun Tu

**Affiliations:** 1 Department of Orthopedic Surgery, E-Da Hospital/I-Shou University, 1, E-Da Road, Kaohsiung City, Taiwan 824 Taiwan; 2 School of Medicine for International Students, College of Medicine, I-Shou University, Kaohsiung, Taiwan; 3 School of Medicine, College of Medicine, I-Shou University, Kaohsiung, Taiwan; 4 Department of Physical Therapy, College of Medicine, National Cheng Kung University, Tainan, Taiwan

**Keywords:** Elbow instability, External joint stabilizer-elbow, Biomechanical outcomes, Clinical evaluation

## Abstract

**Background:**

This study aimed to evaluate the outcome of using an External Joint Stabilizer – Elbow (EJS-E) for persistent elbow instability based on biomechanical experiments and analysis of clinical results.

**Methods:**

An EJS-E was used in 17 elbow instability patients. The median follow-up was 26 months (range, 12–42 months). We evaluated the flexion–extension and pronation-supination movement arcs, visual analog scale (VAS) score, Mayo Elbow Performance Score (MEPS), Broberg and Morrey classification system, and occurrence of complications in these patients. Moreover, construct stiffness and maximum strength tests were performed to evaluate the strength of the fixation techniques.

**Results:**

The final median range of the extension-to-flexion and pronation-to-supination arc of the elbow was 135° (range, 110°–150°) and 165° (range, 125°–180°), respectively. The VAS pain scores were > 3 in two patients. The median MEPS was 90 (range, 80–100 points). Five patients showed signs of grade I post-traumatic osteoarthritis according to the Broberg and Morrey radiographic classification system, while grade II changes were observed in three patients. Complications included axis pin loosening with pin-tract infection in two patients, transient ulnar nerve symptoms in two patients, heterotopic ossification in two patients, and suture anchors infection in one patient. Based on the biomechanical testing results, the EJS-E exhibited higher stiffness and resisting force in varus loading. It was 0.5 (N/mm) stiffer and 1.8 (N·m) stronger than the internal joint stabilizer (IJS) by difference of medians (*p* < 0.05).

**Conclusions:**

Biomechanical and clinical outcomes show that EJS-E via the posterior approach can restore mobility and stability in all patients, thus serving as a valuable alternative option for the treatment of persistent instability of the elbow.

## Background

The treatment of traumatic dislocation of the elbow, complicated by associated fractures and/or extensive soft-tissue injuries, is a challenging task [1, 2]. One of the chief objectives of treatment should entail the provision of sufficient stability to permit early postoperative mobilization, owing to the tendency of the elbow to develop a contracture after injury [2, 3]. Therefore, the surgical management of instability of the elbow should establish congruent reduction with sufficient stability such that joint movement may be initiated soon after treatment [1–3]. Unfortunately, this is not possible in every patient, especially in those with irreparable soft-tissue damage and osteoporotic and comminuted fractures that may result in elbow instability at the time of the initial surgical treatment [3]. Several researchers have advocated for the use of an external fixator for the management of elbow fracture-dislocation and joint instability after extensive contracture release [4–7]. However, despite demonstrating satisfactory clinical outcomes, it is seldom indicated. The clinical indications for hinged external fixation range from acute instability after “simple” elbow dislocation to complex posttraumatic fracture-dislocation [3]. However, hinged external fixators are bulky, have difficult application, and are associated with a reportedly high complication rate of 15–38% [3, 8].

Orbay and Mijares et al. designed an internal joint stabilizer (IJS) for treating instability of the elbow using a temporary Steinmann pin bent and placed through the axis of the ulnohumeral joint and then attached to the proximal ulna [9, 10]. This technique restores elbow stability and permits motion, showing promise for the treatment of patients with severe elbow instability [9, 11]. Herein, we present a simple low-profile External Joint Stabilizer – Elbow (EJS-E) that represents a modified version of the IJS for the management of elbow instability. This device is used temporarily to allow for ligament healing and is intended to be removed directly after 4 to 6 weeks without the need for a secondary surgical procedure. From a clinical outcomes point of view, using an IJS for treating complex elbow instability results in a satisfactory outcome [9–11]. However, EJS-E is a new technique that is not generally acknowledged. Moreover, insufficient stiffness of the EJS-E may pose a concern. Literature describing fixation stability using this technique is limited; thus, clinical recommendations on its practical use in reducing implant failure risk remain to be determined.

This study aims to determine whether an EJS-E provides sufficient stability to maintain the elbow joint, allowing early range of motion and thereby achieving bone and soft-tissue healing. The second goal was to compare the biomechanical stiffness of EJS-E with IJS to demonstrate whether the use of our EJS-E for persistent elbow instability is an optimal alternative.

## Patients and methods

### Biomechanical pilot study

To validate the design feasibility of our innovative EJS-E, the ulna and humerus bone models were used to evaluate the joint stability repaired by the innovative EJS-E and as comparison against a validated IJS. For the biomechanical evaluation, the experimental setup was to simulate a cantilever bending test in the varus direction. The construct stiffness and maximum strength were utilized as key indications for joint stability.

**Fig. 1:**
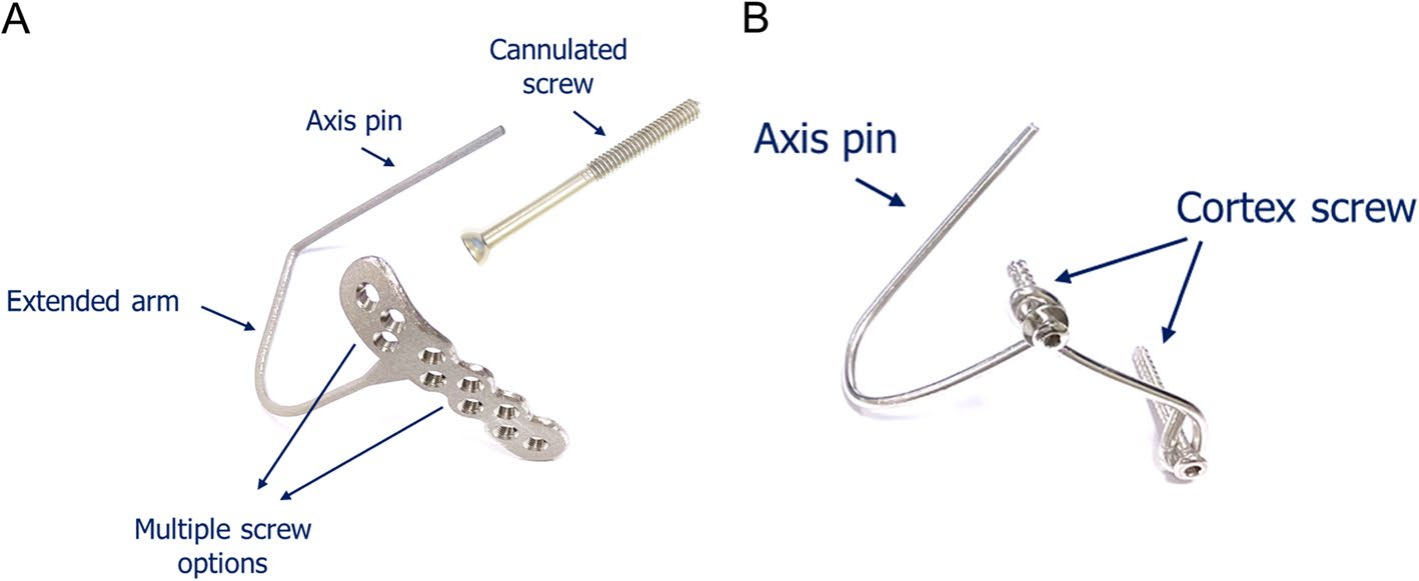
The two different joint stabilizing devices. **A** The external joint stabilizer—elbow. The axis pin can be inserted through the gliding hole of the 3.5-mm cannulated screw. The cannulated screw is used to prevent toggling effect of the axis pin in the bony channel. Additionally, this device has multiple screw holes at the plate body which benefits surgical flexibility for bony anatomy. **B** The Steinmann pin was shaped as a hook and can be attached to the bone by two cortex screws

The authors used 10 reinforced, solid fourth generation composite ulna and humerus (Sawbones Inc). Two different stabilizing devices were tested for fixation of the elbow joint model, and thus the samples were divided into external joint stabilizer – elbow (EJS-E, *n* = 5) and internal joint stabilizer (IJS, *n* = 5) groups. The innovative EJS-E includes a cannulated screw (diameter of 5 mm, 60 and 80 mm in length) and a plate-based design device with a hooked axis pin (diameter of 3 mm, 75 mm in length) which was manufactured from medical-grade titanium alloy (Ti-6Al-4 V ELI) (E-DA, Kaohsiung, Taiwan) (Fig. 1A). The cannulated screw is inserted into the elbow rotational center in the distal humerus. The plate is 17 mm wide and 75 mm long with a thickness of 3 mm with multiple screw holes (10 holes). The distance between the axis pin and the plate is 55 and 75 mm. The plate is fixed onto the proximal ulna and olecranon by 3 or 4 locking screws (diameter of 3.5 mm). The hooked axis pin is designed to be inserted through the cannulated screw and into the flexion–extension axis of the elbow. Therefore, this device can permit active motion of the joint. The other implant is an IJS created by shaping a 3.0-mm Steinmann pin [9] (Fig. 1B). The shaped implant has an axis pin (the straight section) which is inserted into the distal humerus along the axis ulnohumeral rotation. This device is anchored to the ulna bone by two 3.5-mm cortex screws.

**Fig. 2:**
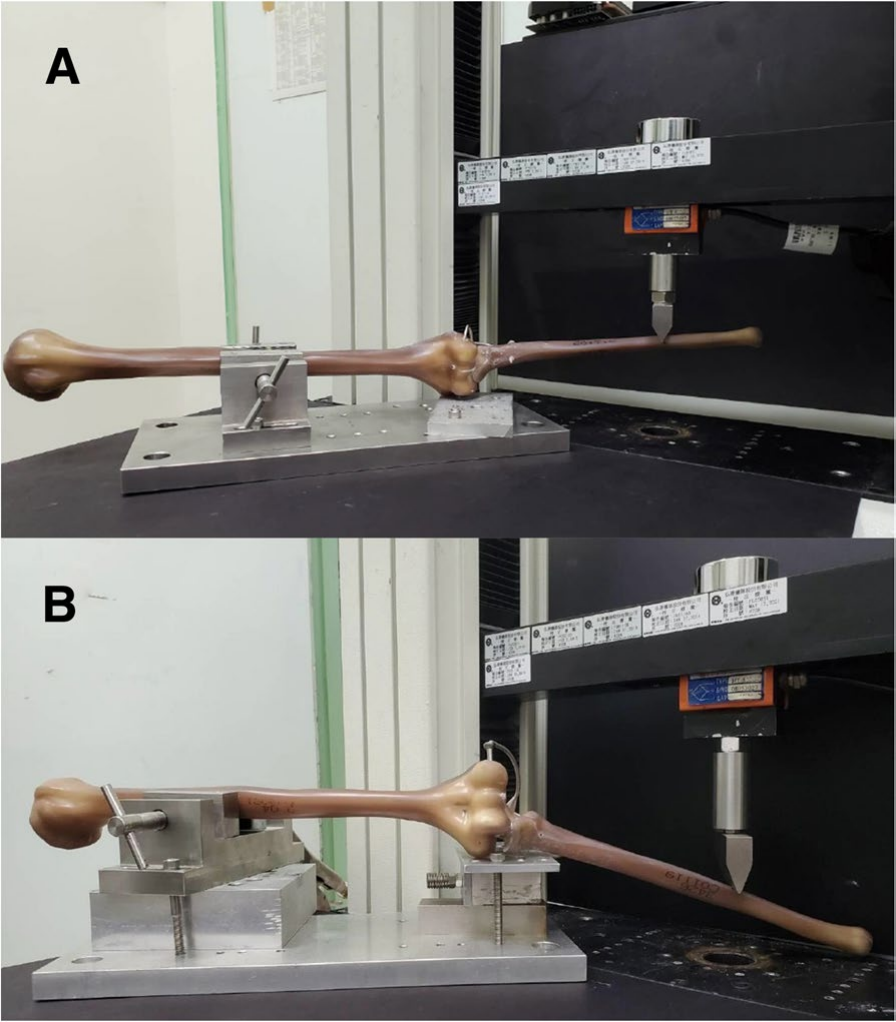
**A** and **B** The experimental setup for biomechanical testing of the external joint stabilizer—elbow and internal joint stabilizer. Axial load was applied on the distal ulna with the synthetic humeral shaft secured by a clamp jig. The distal humerus was supported on a metal block

To prevent soft-tissue effect and minimize interspecimen variations, synthetic humeral and ulnar bones were employed to clarify the pure mechanical properties of the two fixators. The radius was excluded because its presence might affect the construct stiffness and prohibit the identification of the isolated mechanical properties of the joint stabilizers. There was no gap between the humeral and ulnar bones (simple contact), simulating reconstruction [12]. In each specimen, both fixation designs were attached to the lateral side of the ulna. All fixation procedures were performed by one senior orthopedic surgeon (C.-H. Ma). After the specimen preparation, a cantilever bending test was performed using an electric test instrument (Instron Corp., Canton, MA). The test was performed at 0° flexion. The humeral shaft was clamped by a fixture and the medial epicondyle of the distal humerus was supported on a metal block. A compression load was applied to the ulna at a point 150 mm distal to the coronoid process (Fig. 2A and B) [12]. This loading type created a varus moment to the ulnohumeral joint, depending on the orientation of the bones. A 20 N preload was applied to remove any laxity in the structure after which the specimens ware tested to failure at a rate of 1 mm/s. Failure was defined as a 20% drop in load.

During testing, the load and the displacement data were recorded. Construct stiffness against varus moment was calculated from the slope of the linear portion of the load–displacement curve. Maximum strength (the moment) was determined by progressive, increased loading to failure.

### Patients

Seventeen patients with elbow instability were treated with the EJS-E (E-DA, Kaohsiung, Taiwan) at our institute between September, 2017 and August, 2020. The inclusion criteria were sufficient bone quality and quantity to hold the device and encompassed recurrent dislocation or subluxation of the elbow following the management of dislocation or fracture dislocations involving one or a combination of the following: lateral ulnar collateral ligament, medial collateral ligament, radial head, olecranon process, and coronoid process. The patients’ medical records were reviewed retrospectively after obtaining approval from the institutional review board.

The study population included 12 men and five women with a median age of 49 years (range, 27–78 years) (Table 1). Three patients (17.6%) were 65 years of age and older. Five patients (23.5%) had a body mass index of 30 or greater. The mechanism of injury included a fall from a height (5), traffic accident (9), and sports injury (3).

**Table 1:** Patients’ demographic data

Case	Age/Sex	BMI	Injury mechanism	pattern	Close/open	Initial treatment	Fracture (classification)	ORIF	Radial head prosthesis	LCL/MCL	Interval injury to index surgery (weeks)	Concomitant injury
1	51/M	32.4	TA	Monteggia	Close	Non	R(II) O(IIIB).C(II)	R.C.O	-	-/-	1	
2	31/M	25	TA	Dislocation	Close	Splint	-	-	-	+ / +	4	Subarachnoid hemorrhage
3	67/M	29.5	Fall down	TT	Close	K-wire and cast	R(II).C(I)	R	-	±	8	
4	27/F	21.1	Sport injury	Dislocation	Close	Reduction and cast	-	-	-	+ / +	5	
5	70/F	31.6	Fall down	TT	Close	K-wire and cast	R(III).C(II)	C	+	±	4	Rheumatoid arthritis
6	78/M	27	TA	TT	Open	Non	R(II).C(I)	R	-	+ / +	1	
7	49/M	29	TA	Monteggia	Open	Debridement Ext-fx	R(II). O (IIA)	R. O	-	-/-	5	
8	52/M	30.2	TA	TT	Close	K-wire and cast	R(II).C(I)	R	-	+ / +	4	
9	59/M	26	Fall down	TT	Close	Reduction and cast	R(II).C(I)	R	-	+ / +	6	Calcaneus fracture
10	42/F	28.5	TA	TT	Open	Debridement Ext-fx	R(III).C(II)	C	+	±	2	Distal radial fracture
11	32/F	27	Sport injury	TT	Close	LCL-r and cast	R(I).C(I)	R	-	±	4	
12	55/M	29	Fall down	Dislocation	Open	Debridement Ext-fx	-	-	-	±	1	Distal radial fracture
13	45/M	32	Fall down	TT	Close	RH-p, Coronoid-p	R(III).C(II)	C	+	±	2	
14	58/M	25.5	TA	Dislocation	Close	Reduction and cast	-	-	-	+ / +	8	Clavicle Fracture
15	34/M	28	Sport injury	Dislocation	Close	Reduction and cast	-	-	-	±	6	
16	29/M	24	TA	TT	Close	Reduction and cast	R(II).C(II)	R. C	-	±	4	
17	39/F	25	TA	TT	Close	RH-k + cast	R(III).C(I)	-	+	±	4	

*M* Male, *F* Female, *BMI* Body Mass Index, *TA* Traffic accident, *TT* Terrible triad, *Ext-fx* External fixation, *LCL-r* Lateral collateral ligament –repair, *RH-p* Radial head plating, *RH-k* Radial head-K-wire, *Coronoid-p* Coronoid-plating *R* Radial head(Mason classification), *C* Coronoid process(O’Driscoll classification), *O* Olecranon(Mayo classification), *ORIF* Open redaction internal fixation, *LCL* Lateral collateral ligament, *MCL* Medial collateral ligament

Thirteen injures were closed in nature and four were open. Ten patients presented with posterior dislocation of the elbow with fractures of the radial head and coronoid process that were identified as the terrible triad of the elbow, five patients had an unstable joint due to elbow dislocation and two patients had a posterior Monteggia-pattern injury. The indications for the use of the EJS-E included the inability to accomplish complete osseous and ligamentous repair and persistent instability secondary to failure of operative or non-operative management^3^. The initial treatment consisted of surgery in nine patients (including one patient with open dislocation, one patient with posterior Monteggia fracture, and seven patients with terrible triad injuries) and nonsurgical methods (closed reduction and immobilization) in two patients with terrible triad injuries and four patients with elbow dislocation. The median interval between the injury and index surgery for residual instability is 4 weeks.

**Fig. 3:**
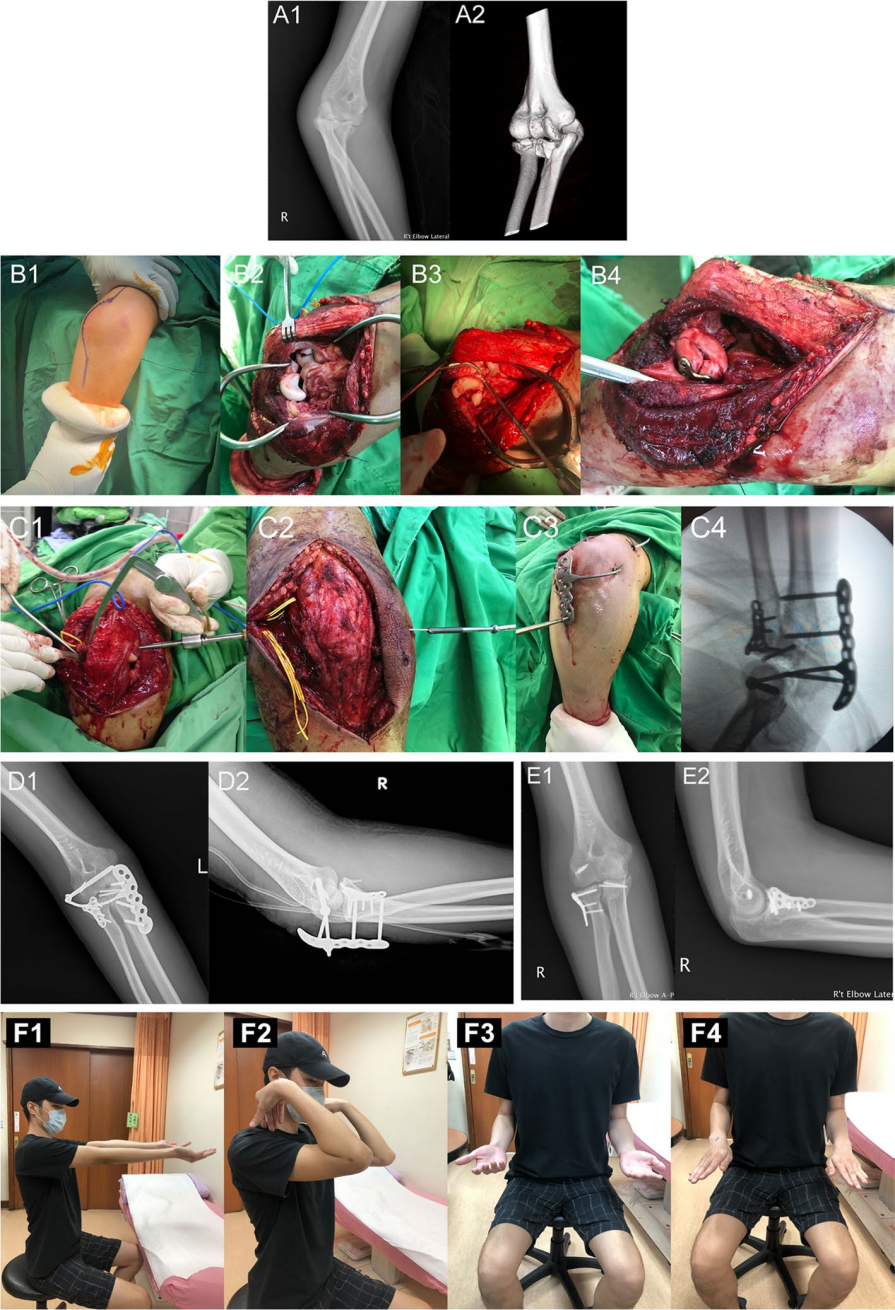
X-ray radiographs, computed tomography (CT) scans, and surgical procedure. (A1, A2) Radiograph and CT scan of a 29-year-old male patient with a right terrible triad of elbow dislocation (patient 16). The surgical photograph shows (B1) elbow exposure via a curved posterior incision, (B2) wide exposure of the elbow joint to reveal the lateral collateral ligament, radial head, and coronoid injury through the lateral or medial margin of the flaps, (B3, B4) radial head and coronoid process fractures treated using a plate and 2.4-mm cannulated screws for fixation. (C1) A line between the origins of both collateral ligaments reveals the axis; a 2.0-mm K-wire using an aiming device from the lateral side into the distal humerus along the axis, (C2) 5.0-mm cannulated screws inserted into the elbow rotational center, (C3) the hooked axis pin is inserted into the cannulated screw, and the external locking plate is fixed with 3.5-mm locking screws over the proximal ulna, (C4) stability in all directions was assessed under fluoroscopic guidance. (D1, D2) Anteroposterior lateral radiographs of the status after open reduction, internal fixation, lateral collateral ligament reattachment, and external joint stabilizer—elbow implantation. (E1, E2) Radiographic images of the elbow 1 year after the final removal of the implant. (F1-F4) Functional range of motion observed at the 1-year follow-up

Plain radiography and computed tomography (CT) were performed to evaluate the osseous abnormalities in all patients preoperatively (Fig. 3A1 and A2). The plain radiographs were acquired in two views 1, 2, 3, 6, 9, and 12 months after surgery. Three-dimensional CT was routinely used in all cases before surgery to identify the fracture patterns, comminution, and displacement, which may not be evident on plain radiographs. This study employed O’Driscoll et al.’s classification of coronoid fractures that categorizes fractures according to their location with reference to the local anatomy on the CT scan [13]. Radial head fractures were classified according to the original Mason classification [14]. Olecranon fractures were classified according to the Mayo classification [15].

### Surgical technique

Routine physical examinations were performed under anesthesia, with the patients placed in the lateral decubitus position. A tourniquet was used in all cases. The elbow was exposed via a curved posterior incision and a global approach was used (Fig. 3B1). Cutting the skin over olecranon and proximal ulna should be avoided for facilitating the subsequent screws insertion. The ulnar nerve was routinely identified, released from its tunnel, and protected. Broad medial and lateral full-thickness soft-tissue flaps were elevated, and the posterior elbow capsule was isolated and excised. Access to the elbow joint was improved by supinating the forearm away from the distal humerus, which enabled wide exposure of the elbow joint for examination of the soft tissue and bone structure injury through the lateral or medial margin of the flaps (Fig. 3B2).

Five patients who sustained a type II coronoid fracture underwent internal fixation (Fig. 3B3). Coronoid process fractures were managed according to O’Driscoll’s classification: Type I coronoid tip fractures did not require fixation and type II and III coronoid fractures were treated using 2.4-mm cannulated screws and/or a 2.4-mm buttress plate for fixation. Twelve patients had accompanying radial head fractures. Eight of these 12 patients were treated with open reduction-internal fixation of the radial head fracture (Fig. 3B4), and four patients were treated with radial head protheses. Olecranon fixation was performed in two patients. Once bony reconstruction was complete, the lateral collateral ligament complex and surrounding soft tissue were reapproximated to the lateral humeral ridge with suture anchors in 15 patients, and the origin of the medial collateral ligament was reattached in six patients with dislocation via suture anchors.

Subsequently, a line between the origins of both collateral ligaments revealed the axis. We drilled a 2.0-mm K-wire using a cruciate ligament reconstruction aiming device and assessed under fluoroscopic guidance from the lateral side into the distal humerus along the axis, passing through the centers of the trochlea and capitellum, to prepare the axis tunnel of the elbow rotational center (Fig. 3C1). We used a 3.8-mm cannulated screw drill to prepare the elbow rotational center. Then, 5.0-mm cannulated screws were inserted into the elbow rotational center and the cannulated screw head stayed outside the skin (Fig. 3C2).

After obtaining concentric reduction and appropriate bone and soft-tissue reconstruction, each patient was fitted with an external joint stabilizer-elbow (E-DA, Kaohsiung, Taiwan). This external stabilizer consists of two components, i.e., a hooked axis pin and external locking plate. The hooked axis pin is inserted into the cannulated screw, and the external locking plate is fixed with 3.5-mm locking screws through stabbing the skin wound over the olecranon and proximal ulna (Fig. 3C3). Before fixation of the external locking plate with locking screws, we temporarily closed the skin wound to avoid uneven distribution of soft-tissue tension that can occur due to screw insertion to the proximal ulna, resulting in difficult wound closure. We inserted 1 or 2 bi-cortical locking screws in the proximal ulna and 1 or 2 uni-cortical locking screws in the olecranon to avoiding into elbow joint (Fig. 3C3). Finally, restoration of elbow flexion/extension, pronation/supination, and stability in all directions were assessed under fluoroscopic guidance before wound closure (Fig. 3C4).

### Postsurgical management

All patients were administered upper limb peripheral nerve blocks for pain control for the first week. Patients wore a protective splint, with the elbow in 90° flexion for the first 2 days postoperatively. Subsequently, the active range of motion (ROM) was instituted as tolerated, while a gentle passive ROM was allowed under adequate pain control. No limit was placed on flexion/extension and pronation/supination movements. Screw-tracks were cleaned with 75% alcohol three or four times daily. The EJS-E was directly removed 4–8 weeks after surgery under local anesthesia at the out-patient follow-up clinic when soft-tissue healing was expected to maintain stability.

Patients were followed up clinically and radiographically until fracture union and until the plateau stage of the range of elbow motion was achieved within at least 1 year (Fig. 3D–F). The function and stability of the elbow joint, pain via visual analog scale (VAS) [16], and incidence of complications were assessed, and the results were recorded. Radiography was used for the identification of any screw loosening or radiolucency around screw, synostosis, heterotopic ossification (HO), and joint congruency. The Broberg and Morrey classification was used for the evaluation of traumatic arthritis [17]. The Mayo Elbow Performance Score (MEPS) was determined for each patient at the final clinic visit. The MEPS measures elbow function across four domains: pain (45 points), stability (10 points), ROM (20 points), and daily functional tasks (25 points). Scores are categorized as 90–100 = excellent, 75–89 = good, 60–74 = fair, 0–59 = poor [18].

### Statistical analysis

Descriptive statistics for the study population were performed to depict patient demographics, injury characteristics, follow-up time frame, and functional outcomes. Due to the small sample size, we assumed that the data was not distributed normally. Therefore, we presented them as the median and range.

Biomechanical testing results were analyzed using SPSS statistical software (SPSS Inc). The difference of construct stiffness and maximum strength between the external joint stabilizer-elbow (EJS-E) and internal joint stabilizer (IJS) were evaluated using the Wilcoxon rank-sum test. Statistical significance was set at *p* < 0.05.

## Results

### Biomechanical testing results

Axis pin deformity of the tested specimens in both the innovative EJS-E and the IJS was observed (Table 2). Based on the biomechanical testing results, the median of construct stiffness of the EJS-E and IJS was 1.4(range, 1.3–2) and 0.9(range, 0.9–1.3) N/mm, respectively. Maximum strength of the EJS-E and IJS was 9.7(range, 9–10.3) and 7.9(range, 6.4–8.5) (N·m), respectively. The EJS-E exhibited higher stiffness and resisting force in varus loading. It was 0.5 (N/mm) stiffer and 1.8 (N·m) stronger than the internal joint stabilizer (IJS) by difference of medians. The statistical analysis results showed that the axial stiffness was significantly different among the groups (*p* < 0.05).

**Table 2:** Biomechanical results

Constructs	**Innovative hinged fixator**	**Steinmann pin stabilizer**	**Difference of medians**	***p* value**
Construct stiffness (N/mm)	1.4(1.3–2)	0.9(0.9–1.3)	0.5	< 0.05
Maximum strength (N·m)	9.7(9–10.3)	7.9(6.4–8.5)	1.8	< 0.05

Data presented as median (range)

### Clinical and radiographic outcome

All patients underwent a satisfactory follow-up for a median duration of 26 months (range, 12–42 months) (Table 3). The median duration of external stabilization was 5 weeks (range, 4–8 weeks). The median ROM at EJS-E removal of the extension-to-flexion and pronation-to-supination arc of the elbow was 120° (80°–145°) and 155° (100°–180°), respectively. The final median range of the extension-to-flexion and pronation-to-supination arc of the elbow was 135° (110°–150°) and 165° (125°–180°), respectively. Ten patients reported a pain score 1 on VAS, three patients a score 2, and two patients a score 3. The median MEPS was 90 (range, 80–100 points) at the final follow-up. This score reflected excellent results in 11 patients, and good results in six patients. Fair or poor results were not detected in any patient.

**Table 3:** Clinical Results

Case	Follow-up (month)	DES (weeks)	Cannulated Screw	ROMAR E/F, P/S	ROMAF E/F, P/S	VAS	B-M	MEPS	Complication
1	42	6	-	110/180	135/180	1	1	90	
2	32	6	-	120/170	145/180	1	0	95	
3	36	4	-	130/155	130/180	3	2	80	Pin tract infection, HO
4	26	6	-	135/180	145/180	1	0	100	
5	32	4	-	100/135	120/135	3	1	80	Pin tract infection
6	36	6	+	120/160	140/160	1	0	100	
7	30	6	+	130/175	150/180	1	0	95	Ulnar nerve
8	30	4	+	110/150	135/170	1	1	90	
9	24	6	+	145/180	145/180	1	0	100	
10	26	5	+	125/180	140/180	1	0	85	HO
11	28	4	+	130/140	145/165	1	0	95	
12	18	8	+	90/100	120/130	2	2	80	Ulnar nerve, HO
13	24	6	+	125/110	115/125	2	0	85	
14	12	4	+	120/100	130/135	1	1	100	
15	18	5	+	110/150	135/160	1	0	90	
16	20	5	+	100/160	125/160	2	1	95	
17	14	4	+	80/120	110/155	1	2	80	Suture anchors infection

*DES* Duration of external stabilizer, *E/F* Extension/Flexion, *P/S* Pronation/Supination, *ROMAR* Range of motion at removal, *ROMAF* Range of motion at final, *VAS* Visual analog scale, *B-M* the Bromberg and Morrey system for elbow osteoarthritis, *MEPS* Mayo Elbow Performance Scores, *HO* Heterotopic ossification

Postoperative radiographs obtained at follow-up revealed concentric, anatomic restoration, without objective signs of instability in all cases. Five patients showed signs of grade I posttraumatic osteoarthritis according to the Broberg and Morrey radiographic classification system, while grade II was found in three patients; grade III was not found in any patient. Complete bony union of all coronoid, radial head, and olecranon fractures treated with internal fixation was achieved. Slight HO was evident in two patients, but neither required additional surgery. Two patients exhibited the stress shielding effect around the axis tunnel of the elbow joint, based on follow-up radiographs obtained before the removal of the external joint stabilizer. Both patients had pin-tract infection of the hooked axis pin, which healed after oral antibiotic therapy and external joint stabilizer removal. One patient had wound infection resulting from suture anchor and required wound debridement. Transient ulnar nerve symptoms developed postoperatively in two patients. This problem resulted from ulnar nerve manipulation during surgery and recovered spontaneously within 3 months.

## Discussion

There is some debate on the risk of persistent instability of elbow fracture-dislocation after operative treatment. In the current study, we found that some instability resulted from that surgeon’s lack of understanding of the pathomechanism of elbow injury, resulting in an inadequate repair strategy. Osteopenia was diagnosed in 50% of the women aged ≥ 65 years by dual-energy X-ray absorptiometry scans in Taiwan [19]. Osteoporosis is an impediment to internal fixation in elderly patients with elbow injury, especially when dealing with small fragments and comminution of the articular surface that may lead to persistent instability and require the adjunctive use of an external fixator [3, 20]. Dislocation or subluxation for as little as 2 weeks causes changes to bone and soft-tissue properties, such that restoration of osseous stability and repair of the collateral ligaments may be inadequate to maintain elbow reduction [11]. Moreover, patients with a higher body mass index may be at risk for residual instability since the elbow experiences higher valgus loading when the shoulder is abducted in these patients [21–23]. Salazar et al. demonstrated that the internal joint stabilizer can be used to successfully augment standard methods of care and regain elbow stability, even in patients with obesity, complex comorbidities, and difficult fracture patterns [23]. The above-mentioned risk factors for residual instability after surgery were also observed in the current study population.

An irreparable comminuted coronoid fracture is considered as a risk factor of persistent instability after elbow injury. Methods to address persistent instability include applying an external fixator or the placement of transarticular pins with casting to maintain the ulnohumeral joint [24–26]. Reiter et al. reported a biomechanical study on cadaveric specimens with O’Driscoll type 2–subtype III coronoid fractures and demonstrated that the internal joint stabilizer provided equal stability to external fixation while against a gravity stress at 60˚ abduction. The authors concluded that an internal joint stabilizer could provide support against the varus posteromedial instability stress. [27] The function of our EJS-E is similar to that of the internal joint stabilizer. Hence, our implants could be considered as an alternative for patients with elbow posteromedial instability or comminuted coronoid fractures.

Allison et al. described the use of static elbow external fixation in cases of complex elbow fracture-dislocation and chronic instability, which resulted in most patients obtaining a stable joint with a functional ROM [6]. The average final arc of flexion–extension was 114°, and average MEPS was 89. In their case series, the external fixators were left in place for an average of 37 days (range, 19–47 days) and five patients (25%) required additional operation for elbow stiffness after fixator removal. Al Qahtani et al. compared static and dynamic external fixation for elbow instability revealing equally effective clinical outcomes [7]. The average final arc of flexion–extension and pronation-supination in the static fixation group was 102° and 138° and in dynamic fixation group was 104° and 131°, respectively [7]. However, the limitation of static fixation is its potential for stiffness [6, 7]. Timing of external fixator removal is crucial for achieving stable joint and avoiding joint stiffness. McKee et al. and Yu et al. reported that hinged external fixation also allowed for immediate motion [4, 5]. The mean final arc of flexion–extension and pronation-supination in McKee et al.’s study was 105° and 151°, respectively and in Yu et al.’s study was 93° and 96°, respectively. The average MEPS was 84 and 75, respectively. Orbay et al. described using a hinged internal Steinmann pin and internal joint stabilizer-elbow for elbow instability [9, 10]. The average final arc of flexion–extension and pronation-supination was 115° and 139° in the Steinmann pin group and 119° and 152° in internal joint stabilizer-elbow group, respectively. The average MEPS was 93 in the internal joint stabilizer-elbow group. Ma et al. reported that the IJS with a standardized treatment protocol could maintain concentric reduction while allowing early functional motion for patients with complex persistent elbow instability. The mean final arc of flexion–extension and pronation-supination was 113° and 148°, respectively. The average MEPS was 95 [11]. In the current study, clinical outcomes were similar to those of the other reported series. All patients were encouraged to perform unsupported motion exercises and were allowed active use of the extremity for light activities of daily living immediately after discharge. The median arc of flexion–extension and pronation-supination at EJS-E removal was 120° (80°–145°) and 155° (100°–180°) and median final arc of flexion–extension and pronation-supination was 135° (110°–150°) and 165° (125°–180°), respectively. The medial MEPS was 90 (range, 80–100 points).

Orbay and Mijares et al. described the use of a temporary Steinmann pin bent and placed through the axis of the ulnohumeral joint and then attached to the proximal ulna as a hinged internal joint stabilizer to treat persistent instability of the elbow [9]. The simple and smart internal device is similar to the hinged external fixator in that it is able to maintain concentric reduction, prevent redislocation, and permit functional motion during the healing period while avoiding problems inherent to other methods of imparting temporary stability. They also translated the idea to develop a new device intended for use as a temporary internal hinged fixator: the internal joint stabilizer-elbow [10]. Although, they described the benefits of avoiding the complications and discomfort associated with an external fixator and its biomechanical advantage, it is not without its drawbacks. The disadvantages of the internal joint fixator include the following (1) the Steinmann pin or 2.4-mm K-wire are too rigid and difficult to be bent; (2) hardware on the olecranon causes friction against soft tissue and results in pain during exercise; (3) seroma formation due to hardware irritation (4) a second operation is required for removing the implant [11]. The EJS-E utilized in the current study is a titanium implant, which includes two components, a hooked axis pin and external locking plate. We used a hooked axis pin inserted into the rotational center of the distal humerus and an external olecranon locking plate instead of attaching a Steinmann pin to the proximal ulna, which provided stability in the coronal plane, while permitting elbow flexion/extension and pronation/supination immediately. This instrument and technique are analogous with the principle that an IJS can prevent redislocation and permit ROM, while decreasing the inherent drawbacks of the temporary stability imparted by the external elbow fixator and IJS, without hardware irritation causing pain, seroma formation and the need for a second surgery for its removal.

Both external fixator and IJS provided sufficient clinical stability [4–7, 9, 11]. From a biomechanical point of view, whether the EJS-E provides appropriate stability to maintain initial elbow stability and allow early rehabilitation remains a concern. In the biomechanical pilot study, an in vitro study, we used the ulna and humerus sawbones elbow model without soft tissues that was unable to accurately mimic the clinical situation and impact of elbow joint kinematics with the stabilizer. We attempted to compare the EJS-E and IJS with construct stiffness and maximum strength. The construct stiffness and maximum strength were utilized as key indications for joint stability. We performed a cantilever bending test using an electric test instrument (Instron Corp., Canton, MA) at 0° flexion, as it the most susceptible position for the dislocation or subluxation of the elbow [12]. The results revealed that the EJS-E exhibited higher stiffness and resisting force in varus loading. We inferred that the EJS-E is biomechanically feasible as an alternative option for elbow instability.

The placement of a hinged external elbow fixator can be demanding [28, 29]. The most critical step is the correct placement of the axis pin. However, accurate pin positioning is a challenging task: the freehand accurate alignment of the hinge to the center of rotation of the elbow or insertion of a provisional pin into the distal humerus under navigation guidance requires a steep learning curve, is time-consuming, and requires additional exposure to radiation [30]. In our surgical protocol, the posterior approach was used via a single curved incision that provided adequate exposure to identify soft tissue and bony structures associated with the lesion. This technique also allows for the protection of the ulnar nerve, reduction or re-alignment of the elbow joint, convenient repair of bony and ligament structures, and application of the EJS-E and is associated with fewer complications related to wound healing. Moreover, we used the open method and a line between the origins of both collateral ligaments revealed the axis [9, 11]. A 2.0-mm K-pin with an aiming device was used for insertion from the lateral side into the distal humerus along the axis under fluoroscopic guidance, passing through the centers of the trochlea and capitellum to prepare the axis tunnel. Because the lateral epicondyle is a relatively easy site from which to define the rotational center [9, 11], it renders the technique more reliable for the acquisition of an accurate axis of rotation.

Despite the growing recognition of the value and indications of hinged external fixators of the elbow, the drawbacks associated with its application cannot be ignored, including pin-tract infection, pin loosening, loss of reduction, risk of fracture, injury to the adjacent neurovascular structures, bulkiness, and limitation of elbow pronation-supination [3, 8]. However, the EJS-E can also prevent or decrease the incidence of these undesirable problems. It is a simple, lightweight, and low-profile device that can be placed during treatment. Theoretically, it also confers a biomechanical advantage over the hinged external fixator as it is more forgiving of small deviations from the exact axis of rotation because the distance from the humerus and ulna to the plate is considerably lesser than that in a hinged external fixator [9, 11]. It incorporates a fixed-angle locking plate device instead of the pin and rod system used by conventional external fixators. Studies that investigated the use of the locking plate as an external fixator for the fixation of extremity fractures stated that it is a reliable option that provides adequate stability for bone union and satisfactory outcomes [31]. The EJS-E does not require the insertion of a pin into the humeral and ulnar shaft through soft tissue; thus, it can bypass issues such as injury to the muscle–tendon unit and adjacent neurovascular structures [3, 8, 29]. The application of the EJS-E allowed full ROM of the elbow, without hindering its pronation-supination.

Pin-tract infection and loosening were inevitable complications of the external fixator [3, 8]. The EJS-E has an increased chance of pin-tract infection and loosening in the rotational center of the distal humerus, particularly in heavier patients and more osteoporotic bone. In this study, the hooked axis pin of the EJS-E was directly inserted into the rotational center in the initial five patients, and two of them revealed rotational center pin-tract infection and stress shielding effect around the axis tunnel of the elbow joint. The problems may result from increased transfer of frictional stress to the pin-bone interface of the rotational center in the soft cancellous bone of the distal humerus, abnormal joint kinematics, and/or incongruous articulation, resulting in a stress shielding effect around the axis tunnel and tract infection. Therefore, we inserted additional cannulated screws into the rotational center of the distal humerus and then the hooked axis pin of the external joint stabilizer into the cannulated screw hollow that shifted the frictional stress to the pin-cannulated screw interface to reduce the risk of pin-tract infection and loosening. In addition, the external locking plate was placed onto the proximal ulna. There are many screw holes in the locking plate (10 holes) and using three or four locking screws for fixation with even distance to each other provided adequate stability.

This study has several limitations. First, we had relatively few patients in this series, which means that there could be complications that would have been observed in larger series that did not occur here. Second, our study has the potential for assessment bias. The preoperative condition and postoperative outcomes were recorded by operating surgeons and ascertained by chart review. Generally speaking, surgeons overestimate the benefits and underreport complications, which makes treatments look better than they really are. Third, the current study is its retrospective design without control group which did not indicate EJS-E available for all type instability of elbow.

In conclusion, biomechanical and clinical outcomes show that EJS-E via the posterior approach can restore mobility and stability in all patients, thus serving as a valuable alternative option for the treatment of persistent instability of the elbow. Further biomechanical studies and prospective clinical trials are required to evaluate the validity of the EJS-E for the treatment of these challenging injuries.

## Data Availability

The datasets used and analysed during the current study are available from the corresponding author on reasonable request.

## Abbreviations

EJS-E: External Joint Stabilizer-Elbow
IJS: Internal joint stabilizer
VAS: Visual analogue scale
MEPS: Mayo Elbow Performance Scores
HES: Hinged external stabilizer
HIS: Hinged internal stabilizer
CT: Computed tomography
ROM: Range of motion
HO: Heterotopic ossification
